# Mental Maladies

**Published:** 1845-08

**Authors:** 


### BIBLIOGRAPHICAL NOTICES.

*Mental Maladies. A Treatise on Insanity.* By E. ESQUIROL, Physician-in-Chief of the Maison Royale des Aliénés De Charenton, formerly Inspector General of the University, Member of the Royal Academy of Medicine, etc. Translated from the French, with additions, by E. K. HUNT, M. D. 8vo. pp. 496. Lea and Blanchard; Philadelphia, 1845.

The writings of Esquirol on Insanity, have long been regarded as of the highest authority, and we rejoice that the more important of them are now republished in this country, rendered into English, and thus placed within the reach of all who may desire to become acquainted with the opinions and experience of that able and estimable writer, on subjects to the investigation of which his long life has been devoted. To quote his own language: "The work now offered to the public, is the result of forty years study and observation. I have noticed the symptoms of insanity, and have studied the manners, habits and wants of the insane, in the midst of whom I have passed my life. I have also tried the best modes of treatment. Confining myself to facts, I have arranged them according to their relations. I have stated them as they have been observed, without, in general, attempting to explain them; and have avoided systems, which always appeared to me more seductive by their splendor, than useful in their application." Accordingly, we find the work throughout to be eminently plain and practical in its character. The materials of it have been collected at the great institution of Salpêtrière, at the Hospital at Charenton, and from his private practice—sources far greater probably than ever were enjoyed by any other individual.

The contents of the volume are arranged under the following heads, viz.: *Insanity; Hallucinations; Illusions of the Insane; Fury; Mental Alienation of those recently confined, and of Nursing Women; Epilepsy; Critical terminations of insanity; Lypemania, or Melancholy; Demonomania; Suicide; Monomania; Mania; Dementia; Idiocy.*

"The first chapter, entitled Insanity, is a summary of the sentiments prevalent on this subject; the remaining ones are commentaries, and a more full exposition of these views."

We shall not presume to criticise the opinions or the practice of an author whose opportunities for observation have been so great, and whose ability to appreciate what he witnessed has been acknowledged by all who have read his productions; but content ourselves with such extracts as will enable those of our readers who are unacquainted with the writings of the author, to judge something of his remarkable acuteness in the perception of facts and resemblances, the closeness with which he has adhered to these in his reasonings, and the clear, concise, and most appropriate language in which his views and observations are set forth.

Under the general head of Insanity, after some very eloquent and feeling remarks on the various manifestations of mental alienation, the claims of the sufferers on our kindness and attention, and for which "an approving conscience is the chief reward," M. Esquirol proceeds to consider his subject under four principal heads, viz.: 1. The symptoms which characterize *insanity*; 2. Its causes; 3. Its progress and different terminations; 4. The general principles of its treatment.

I. *Symptoms of Insanity.*

"Insanity, or mental alienation, is a cerebral affection, ordinarily chronic, and without fever, characterized by disorders of sensibility, understanding, intelligence and will—I say ordinarily, because insanity is sometimes of brief duration; and because, at its commencement, and sometimes during its course, febrile symptoms are manifested. Among the insane, sensibility is exalted, or perverted; and their sensations are no longer in relation with external or internal impressions. They seem to be the sport of the errors of their senses, and of their illusions. Many insane persons do not read, because the letters appear to be mingled in a confused mass, so that they are unable to arrange them in such a manner as to form syllables and words. A thousand illusions of sight, produce, and continue their delirium. They recognize neither their parents nor friends, and regard them either as strangers or enemies. They are no longer correct in the appreciation of the qualities and properties of surrounding objects; many believing themselves at their usual places of abode, when, indeed, they are very far removed from them, and reciprocally."

These various symptoms are illustrated by the recital of cases, with apposite remarks, in the briefest but most explicit language.

II. *Causes of Insanity.*

"The causes of mental alienation are as numerous, as its forms are varied. They are general or special, physical or moral, primitive or secondary, predisposing or exciting. Not only do climates, seasons, age, sex, temperament, profession and mode of life, have an influence upon the frequency, character, duration, crises, and treatment of insanity; but this malady is still modified by laws, civilization, morals, and the political condition of the people. It is, also, produced by causes, whose influence is more immediate, and easily appreciated."

The opinions of our author on the general causes of insanity, are briefly as follows:

1. *Climate*. He regards the predisposition greatest in "temperate climates, subject to great atmospheric vicissitudes, and especially those whose temperature is alternately cold and humid, damp and warm. We see less of insanity in the Indies, in America, in Turkey and Greece; more of it in the temperate climates of the north."

2. *Seasons*. In the Salpêtrière, during nine years, the admissions were most numerous during the months of May, June, July and August; the proportion decreasing from September to December, and still more from February to March.

3. *Age*. "To determine what period of life furnishes the greatest number of insane persons, it was sufficient to bring together the records, made up under very different circumstances. One of them was made at the Bicêtre, where poor men only are received; another at the Salpêtrière, a hospital destined for poor women. The last related to an establishment devoted to the wealthy. From these reports we may conclude: 1st, that the age which furnishes the greatest number of insane, is for men, that from thirty to forty years; whilst for women, it is that from fifty to sixty years; 2d, that the ages which furnish the least, are for both sexes, childhood, youth, and advanced age; 3d, that among women, insanity appears earlier than among men, indeed from twenty-nine to thirty years of age; 4th, that the rich are afflicted, in comparison with the total number of insane persons, in a greater proportion than the poor.."

4. *Sex*. "Cœlius Aurelianus assures us, that women are less subject to insanity than men; and what was true in his time is still so in Italy and Greece. In the north of France the contrary is true; the number of insane women being, in that region, greater than that of men. In England, the number of insane men bears a more equal proportion to that of women. We find the reason for this difference, in the comparison of their habits.

"The vices of education adopted by our young ladies, the preference given to acquirements purely ornamental, the reading of romances, which gives to the intellect a precocious activity and premature desires, together with ideas of an imaginary excellence that can never be realized; the frequenting of plays and society; the abuse of music, and the want of occupation; are causes sufficient to render insanity most frequent among our women.

In England, women receive a more substantial education; they lead a more retired life, and do not take so important a part in public affairs. The social existence of men does not depend so much upon their acts or caprices, and hence there are less insane women than in France."

In summing up the reports, however, from various institutions in different countries, our author arrives at the following general conclusions :

"1. That in a very considerable number of insane people, collected from different countries, and in different conditions, the disparity in numbers between men and women, is much less considerable than is usually supposed :

"II. That this difference approaches very nearly the proportion which exists between the two sexes, in the general condition of the population :

"3. That this difference is not the same in all countries :

"4. That in France the proportion of women is much greater than in England."

5. *Temperament.* "Simple temperaments," the author remarks, "are so rarely met with in practice, that it is not easy to point out with precision, that of this or that individual; and for a still stronger reason, that of one insane person or another. The sanguine temperament constitutes one of the predispositions to mania. The nervous temperament characterized by a susceptibility which every thing irritates and exasperates, in consequence of a susceptibility which deprives its possessor of the faculty of reasoning, is favourable to the production of mania and monomonia."

"In general, those who have black hair, who are strong robust, and of a sanguine temperament, are, when effected, maniacs, and furious. The course of their insanity is more acute, its crises more marked, than among those composing the other classes. Those whose hair is of a flaxen colour, who have blue eyes, and a lymphatic temperament, become maniacs and monomaniacs; but their insanity passes readily into a chronic state, and degenerates into dementia. Those who have black hair and eyes, and who are of a dry, nervous temperament, are more frequently lypemaniacs. Those who have red hair, are furious, traitorous and dangerous."

6. *Profession, and mode of life.* We cannot deny to our readers some of the author's remarks on this division of his subject. The extensive observation of the operations of the human mind which they manifest, the deep reflection, the profound philosophy they inculcate, characterize them more eminently than the productions of any other writer on the same subject with which we are acquainted.

"Persons who devote themselves very perseveringly to study, who abandon themselves to the vagaries of their imagination, who fatigue their intellect, either by a restless curiosity, or by turning aside in obedience to theories and hypotheses, or the allurements of speculative ideas, present a condition favourable to the developement of mental alienation.

"Some, possess an uncontrollable mental mobility; glance at everything, but are incapable of thoroughly investigating any thing. Others, take an interest only in certain objects, and manifest an obstinate tenacity for the same meditations and conceptions.

"These two classes, placed at opposite extremes, stand upon the confines of insanity, unless they constantly keep in check those native dispositions.

"Dryden has said that men of genius, and the insane, stand near together. If he meant by this, that men, who possess very active and disorderly imaginations, who have great exaltation and mobility of ideas, present striking analogies with the insane, he spoke correctly. But if he meant, that great intellectual capacity, occasions a predisposition to insanity, he is mistaken. Men of the greatest genius, both in the sciences and arts, the most illustrious poets, the most skilful painters, have preserved their reason, even to extreme old age. If we have seen painters, poets, musicians, and artists become insane, it is because they associate, with a very active imagination, great errors in regimen, to which their organization exposed them, more than other men. It is not because they exercise their minds, that they lose their reason; nor is it the culture of arts and letters, that we are to accuse. Men, who are endowed with great power of thought and imagination, have special need of sensations. The greater part of painters also, of poets and musicians, impelled by the need of emotions, abandon themselves to numerous errors of regimen; and it is these, far more than excessive study, which are the true cause of their insanity.

"In other cases, the understanding takes an exclusive direction; and the man meditates without cessation, upon subjects connected with metaphysical speculations, and confines himself to them, with a determination proportionate to the efforts that are made to divert his mind. All his physical and moral faculties are absorbed. He neglects the most important personal attentions, condemning himself to practices which seriously affect his constitution."

"The prevailing sentiments of every age, exercise a powerful influence, over both the frequency and character of insanity. It would seem that certain minds, impressed with new conceptions, cannot divest themselves of them. The same effect, which reflection, too long continued, produces upon individuals, is produced upon an entire population. Thus, historical monuments prove, that at the birth of christianity, there was much religious melancholy.

"The chivalrous spirit which succeeded the crusades, multiplied erotic melancholy. Civil and religious discords, excited by Calvinism, caused a return of religious melancholy. Magic and sorcery have had their turn. Ideas of liberty and reform have turned many heads in France, and it is remarkable, that those forms of insanity which have appeared within the last thirty years, have been characterized by the political convulsions, that from time to time have visited our country.

"Finally, it is not discoveries, nor is it a new institution, which always produces insanity."

"In France, there is less insanity in the country, than in cities.

"The countrymen are more apt to contract religious, or erotic insanity. Among them, insanity is caused by the simple passions, love, anger, and domestic chagrin; whilst in cities, it is produced by wounded self-love, disappointed ambition, reverses of fortune, etc.

"The less depraved morals of the Anglo-Americans, are one of the causes, in consequence of which there is less insanity among them than elsewhere; as the reports of travellers prove, not less than the records of their own hospitals.

"In England, where we find united all the caprices, as well as the excesses of civilization, insanity is more frequent than any where else. Unsuitable marriages; those contracted by parents, and above all, alliances formed with families where there is an hereditary predisposition to insanity, the hazards of remote speculations, the indolence of the rich, and the habitual use of alcoholic drinks, are the causes which multiply insanity in England. 'Every thing degenerates in the hands of man,' said J. J. Rousseau. Without doubt, civilization occasions disease, and augments the number of the sick, because, by multiplying the means of enjoyment, it causes some to live too well, and too fast. But the more perfect civilization becomes, and the more the life of the common people is ameliorated, the greater will be its medium duration. Moreover, it is not civilization that we are to accuse, but the errors and excesses of all sorts, which it enables us to commit.

"The morals of the Italians, render religious melancholy and erotomania, very frequent in Italy.

"The ignorance of the middle ages, caused demonomania and vampirism to multiply, and they are now reunited in the extreme north of Europe, as well as in other countries which civilization has not illuminated with its light, nor enriched by its benefits.

" For thirty years, the changes that have been going on in our morals in France, have produced more insanity than our political troubles. We have exchanged our ancient usages and opinions, for speculative ideas, and dangerous innovations.

" Religion comes in only as a usage in the most solemn acts of life,—it no longer brings consolation and hope to the unfortunate. Religious morality no longer guides the reason, in the straight and difficult paths of life :—A cold egotism has dried up all the sources of sentiment. There is no more domestic affection, nor respect, nor love, nor authority, nor reciprocal dependencies. Each lives for himself; no one forming those wise combinations, which connect the present, with coming generations.

" The ties of marriage are mere pretences, which are formed by the wealthy, either as a speculation, or to gratify their self-love; and which the common people neglect, through disdain for the clergy, indifference and libertinism. These deplorable facts, have prevented me from taking an account of the marriage state, of celibacy, or widowhood, among women who enter our hospitals, and consequently, from being able to appreciate, among them, the influence of marriage in the production of mental alienation.

" About one-fourth of the persons admitted into my establishment were bachelors; twenty-six only, were widowers. Having been concerned in the treatment of many soldiers, as well as students, this proportion of single men, of the higher class, will not be surprising.

" The change in our morals, will be felt longer, in proportion as our education is more defective.

" We take great care to form the mind, but seem to forget that the heart, like the mind, has need of education.

" The ridiculous and deplorable tenderness of parents, subjects to the caprices of infancy, the reason of mature age.

" Each gives to his son an education superior to that which is suited to his social position and fortune; so that children, despising the knowledge of their parents, disdain the reproofs of their experience.

" Accustomed to follow all his inclinations, and not being habituated by discipline, to contradiction, the child, having arrived at maturity, cannot resist the vicissitudes and reverses by which life is agitated. On the least adversity, insanity bursts forth; his feeble reason being deprived of its support, while the passions are without rein, or any kind of restraint.

" When we add to these causes, the manner of life of the women in France, the insatiable relish which they have, for romances and the toilette, for frivolities, etc., together with the misery and privations of the lower classes, we shall no longer be astonished at the disorder of public and private morals, nor any longer have a right to complain, if nervous disorders, and particularly insanity, multiply in France; so true is it, that whatever relates to the moral well-being of man, has always a most intimate connection with his physical well-being and the preservation of his health.

"We believe also with Pinel, that an undue severity,—that reproaches for the slightest faults, that harshness exercised with passion, that threats and blows exasperate children, irritate youth, destroy the influence of parents, produce perverse inclinations, and even insanity; especially, if this severity is the result of the caprices and immorality of fathers.

"This system of severity, is less to be feared at this day, than that of which we have spoken above, particularly among those in easy circumstances, and the wealthy.

"The depravations of both minds and morals, which are effected by the vices of our education, by disdain for religious beliefs, and by the faultiness of public morals, exercises its influence, upon all classes of society.

"But how happens it, that we never cease to declaim against the higher class, and to extol the virtues of the people!

"These philosophical declaimers, lived with the great whom they calumniated, and knew not the people. If they had studied the morals of their country, they would have been convinced that the corruption is most general, greatest, most hideous, among the lower class; that it gives birth to almost all the evils of society; that it produces much insanity, and at the same time much more of crime than in the higher classes.

"The vices of education in the higher classes, and the want of it in the lower, explain these differences. Education supplies the place of morals among the former; while no motive suspends the arm of the mob."

7. *Passions.*—Under this head, interesting as it is, and ably handled, our extract must be brief.

"In my *Dissertation on the passions, considered as causes, symptoms and curative means of mental alienation*, I have principally considered them, as the most essential symptoms, and the most powerful therapeutic agents in insanity.

"The first wants of man, limiting themselves to those connected with his preservation and reproduction, provoke the determinations of instinct; an internal impulse leads us to gratify them.

"The secondary wants attach themselves to the first, and the desires which they excite, acquire as much more energy, as we have means of satisfying them. They produce the primitive passions; in fine, they are the wants which are connected with our preservation; and are the fruit of our increased intelligence and civilization. They engender the factitious passions,—those passions which cause the greatest injury to man, especially in the higher walks of life.

"Infancy, exempt from the influence of the passions, is almost a stranger to insanity; but at the epoch of puberty, the sentiments, unknown until this period, cause new wants to arise. Insanity then appears, to trouble the first moments of the moral existence of man.

"At mature age, the relations become extended, social wants multiply, and the passions take a new character. In proportion as the amorous passions become enfeebled, those of a factitious nature grow strong. Personal interest, ambition, love of distinction, and avarice, replace the charms of love and delights of paternity.

"At this period of life also, mental alienation appears; insanity is more obstinate, and more concentrated. It passes more readily into a chronic state; and is more dependent upon abdominal lesions.

"A sense of his weakness renders the old man more calm; and while meditating upon the errors to which the passions lead, he isolates himself, and becomes an egotist.

"Insanity from a moral cause, rarely exists with him, and when he loses his reason, it is because his organs are fatigued and exhausted. Hence, it is neither mania nor monomania which is developed at this period, but senile dementia.

"Of all moral causes, those which most frequently produce insanity, are pride, fear, fright, ambition, reverses of fortune, and domestic trouble. This last should have been placed, relative to its great influence, at the head of moral causes, if it be limited to a simple idea; but by domestic troubles, I express all the pains, all the griefs, all oppositions, misfortunes and dissentions, that grow out of the family state."

We have made more copious extracts from this important work than is our custom, not with the hope of doing the author justice, but that our readers may see something of its excellence, and be induced to buy and read it. Of all the works that have ever been written on the subject of insanity, it is unquestionably the best. The multitude of facts which it contains, briefly and perspicuously stated, and admirably classified; the benevolence and sound christian philosophy discoverable in every page and in every paragraph; the beauty and terseness of its style, and freedom from technicalities—altogether, it is one of the most agreeable books to read, whether for the physician, the lawyer, the divine, or the layman, that has ever come from the press.

In regard to the labors of the translator and editor, we have only room to say, that his translation is creditable, while his occasional notes and comments give additional interest to the work, especially as they express the views at present prevailing on the several topics on this side of the Atlantic.

